# Production of Agarose-Hydroxyapatite Composites via Supercritical Gel Drying, for Bone Tissue Engineering

**DOI:** 10.3390/molecules29112498

**Published:** 2024-05-25

**Authors:** Alessandra Zanotti, Lucia Baldino, Stefano Cardea, Ernesto Reverchon

**Affiliations:** Department of Industrial Engineering, University of Salerno, Via Giovanni Paolo II, 132, 84084 Fisciano, Italy; azanotti@unisa.it (A.Z.); lbaldino@unisa.it (L.B.); ereverchon@unisa.it (E.R.)

**Keywords:** bone, agarose, scaffold, supercritical drying, mechanical properties

## Abstract

Bone tissue engineering (BTE) is the most promising strategy to repair bones injuries and defects. It relies on the utilization of a temporary support to host the cells and promote nutrient exchange (i.e., the scaffold). Supercritical CO_2_ assisted drying can preserve scaffold nanostructure, crucial for cell attachment and proliferation. In this work, agarose aerogels, loaded with hydroxyapatite were produced in view of BTE applications. Different combinations of agarose concentration and hydroxyapatite loadings were tested. FESEM and EDX analyses showed that scaffold structure suffered from partial closure when increasing filler concentration; hydroxyapatite distribution was homogenous, and Young’s modulus improved. Looking at BTE applications, the optimal combination of agarose and hydroxyapatite resulted to be 1% *w*/*w* and 10% *w*/*v*, respectively. Mechanical properties showed that the produced composites could be eligible as starting scaffold for BTE, with a Young’s Modulus larger than 100 kPa for every blend.

## 1. Introduction

Bones are complex structures that perform crucial functions in the human body, such as the protection of inner tissues, locomotion, mineral storage, etc. [1,2]. They show a highly hierarchical morphology, ranging from the macro- to the nanoscale, and a highly multifaceted composition, consisting of both organic and ceramic species (i.e., calcium, phosphorous, collagen, etc.) [3,4]. Traumatic injuries and several diseases can cause bone damage and consistent defects, thus affecting their correct functioning [5,6,7].

Bone tissues can regenerate themselves spontaneously to repair small defects (i.e., <6 mm [8]); however, when injuries or defects fall out of this range, tissue replacement (autograft or allograft) is generally required to recover the damage [9]. Even though bone transplantation is a common practice, it comes with non-negligible drawbacks: autografts suffer from tissue unavailability, whereas allografts lead to immunogenic responses and inflammation, and, eventually, to tissue rejection [10,11]. These limitations can be overcome using bone tissue engineering (BTE) that evolves around three main pillars: scaffolds, cells, and growth factors [11,12].

Scaffolds are 3D porous materials that should temporarily serve as a substitute for the damaged tissue while promoting cell adhesion, proliferation, and differentiation (i.e., new tissue formation) [13]. Moreover, scaffolds must be biocompatible and biodegradable to avoid tissue inflammation and rejection. In addition to these requirements, scaffolds should mimic the original tissue to be replaced in terms of morphology (i.e., macro- and nano-structure should coexist), porosity, mechanical properties, and composition; moreover, in the field of BTE, scaffolds should promote osteogenic differentiation [14,15,16,17]. Following this line of thought, engineered scaffolds can be tailored to meet all these specifications.

Scaffolds can be produced following different routes (e.g., solvent casting and particulate leaching, melt molding, gas foaming, electrospinning, 3D-printing, etc. [18,19,20]), among which the sol–gel technique stands out for its simplicity and repeatability [21,22,23]. The sol–gel route is initiated by the preparation of the sol phase (i.e., a solid suspension in a liquid medium); then, the sol undergoes a gelling step (i.e., the gel is formed) [24]. The scaffold is obtained once the liquid phase is removed from the gel network, and a drying step must be performed. Conventional techniques (freeze-drying and room pressure drying) result in collapsed structures and the low porosity of the sample, which affect the scaffolds suitability for BTE applications [25,26]. Supercritical carbon dioxide (SC-CO_2_) assisted drying—which produces aerogels (highly porous and lightweight materials)—is an efficient strategy that meets the need for a nanostructured network suitable for cell adhesion and proliferation [27,28] due to SC-CO_2_’s zero surface tension and gas-like diffusivity, avoiding structure collapse [29].

A wide range of materials (e.g., chitosan, agarose, alginate, starch, polylactic acid, polyglycolic acid, polycaprolactone, etc.) were explored to produce scaffolds for BTE applications, due to their high biocompatibility and ability to emulate bone morphology [30,31,32,33]. In particular, agarose (AG) is a natural biopolymer extracted from red algae, whose fundamental monomeric unit is agarobiose, a disaccharide made up of D-galactose and 3,6-anhydro-L-galactopyranose. Agarose is drawing attention in the field of tissue engineering because of its gelation behavior, high biocompatibility, ability to mimic bone extracellular matrix (ECM) and promote cell proliferation [34,35,36,37]. Looking at BTE applications, hydroxyapatite (HA) is a major inorganic component of bone, and it is crucial for bone tissue regeneration since it stimulates growth factors and alkaline phosphatase, on top of improving tissue strength and mechanical resistance [29]. Therefore, the addition of HA as bioactive filler to the scaffold is important for tissue regeneration and final performances.

Some attempts to produce agarose-based scaffolds are reported in the literature. Kazimierczak et al. [37] prepared a chitosan/agarose blend with the addition of hydroxyapatite (HA) by freeze-drying. The produced scaffold resulted to be biocompatible and osteoconductive, due to the addition of nano-HA; even though porosity was bound only to the microscale, the composite proved to be effective with respect to bone tissue regeneration, being cellular response active. Wizler et al. [38], using the freeze-drying and SC-CO_2_ drying techniques, obtained AG–HA composites for drug delivery. Even though these authors did not test these composites for BTE purposes, they proved that the AG–HA scaffold was not cytotoxic; moreover, supercritically dried gels showed specific surface areas (SSA) up to an order of magnitude greater than the freeze dried ones: 144 m^2^/g vs. 24 m^2^/g for pure agarose. This trend was observed when HA was added to the polymeric solution as well, even though the specific surface area (SSA) decreased in both cases, increasing filler content. In addition, SEM images showed that SC-CO_2_ dried gel morphology was open and regular also on the nanoscale; whereas freeze dried gels were collapsed and irregular on the microscale, and completely collapsed on the nanoscale. Also, mechanical properties must be assessed for BTE purposes, even though little information is available in the scientific literature. Khanarian et al. [39] explored the effect of the addition of HA on agarose hydrogels. In this case, a 2% *w*/*w* AG scaffold combined with a 6% *w*/*v* micro-HA content resulted in a maximum Young’s modulus (E) of 4.3 ± 0.2 kPa. It is expected that dried structures behave differently from the native hydrogel; however, this result proves that AG–HA formulations should be mechanically improved with a view to BTE.

In light of these considerations, the aim of this work is to produce, for the first time, AG–HA composites using SC-CO_2_ assisted drying, to create a porous structure to host the ceramic filler. Several combinations of agarose and hydroxyapatite will be characterized in terms of morphology, chemical composition, and mechanical properties, to identify the optimum one for bone tissue regeneration.

## 2. Results and Discussion

### 2.1. Bulk Properties

In this work, experiments were organized in two phases: the first one was aimed at producing aerogels of pure agarose (i.e., 1% and 4% *w*/*w*), to prove that SC-CO_2_ assisted drying leads to comparable results with respect to previous works [40]; the second one was based on the production of AG–HA composites. In this latter case, AG quantity was set either at 1% *w*/*w* or 4% *w*/*w*, whereas HA content was changed (namely, 1, 5 and 10% *w*/*v* with respect to the solution volume for both agarose concentrations). As far as the first step is concerned, both 1% *w*/*w* and 4% *w*/*w* aerogels were produced: samples did not shrink significantly after processing, and their bulk properties were monitored over time to evaluate shrinkage phenomena and, thus, their stability. The results of these measurements are reported in Table 1.

Both samples were stable over time overall after a small reduction after 10 days, as shown in Table 1; this information could be useful for storage purposes. Once pure agarose stability was assessed, the bulk properties of AG–HA composites were explored. Table 2 indicates AG–HA composites bulk density and porosity measured post drying.

Expectedly, the composites’ bulk density increased when larger HA concentrations were used, due to having a higher HA density than pure agarose. As far as porosity is concerned, the composites are always less porous than the pure agarose aerogel. The data collected for 1% *w*/*w* AG composites show that porosity ranged from 96.5% to 86.5% with increasing HA concentration; the same trend could be seen for 4% *w*/*w* AG composites, for which porosity moves from 88.5% to 82.1%. Overall, it was proven that the higher the HA percentage in the scaffold, the lower its porosity.

### 2.2. FT-IR Analysis

**Figure 1:**
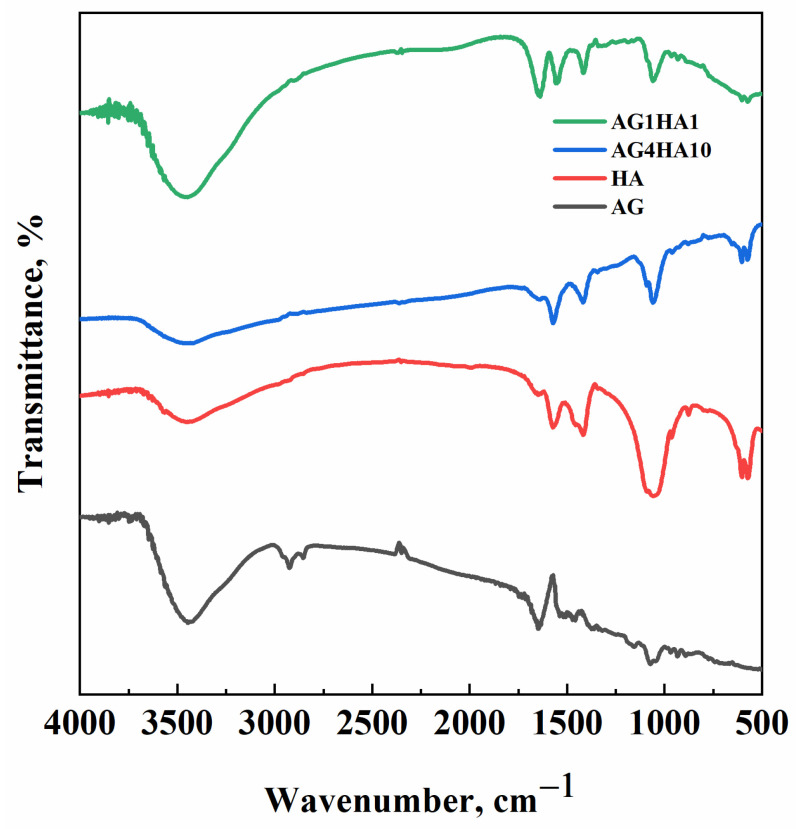
FT-IR spectra of agarose (black), hydroxyapatite (red), AG4HA10 (blue) and AG1HA1 (green).

FT-IR analysis was aimed at highlighting the most significant bonds formed in the composites. Specifically, spectra were collected for the samples AG1HA1 (when agarose and hydroxyapatite contents were comparable) and AG4HA10 (when hydroxyapatite outweighs agarose). In previous works [41], it was proved that SC-CO_2_ drying did not affect agarose chemistry; therefore, in the present research, only agarose aerogel was analyzed as a reference. Figure 1 reports the obtained spectra.

Pure agarose aerogel can be associated with some characteristic peaks: O–H stretching vibration was present at about 3400 cm^−^^1^, whereas its bending vibration emerged at 1645 cm^−^^1^; C–O stretching was located around 1100 cm^−^^1^. The peaks observed in the fingerprint region (i.e., 500–1000 cm^−^^1^) were associated with the C–H bending vibrations of the 3,6-anhydrogalactose structure [42]. The HA spectrum was similar to the ones reported in the scientific literature [43]. The phosphate groups (PO_4_^3−^) showed typical peaks at around 1000 cm^−^^1^ and 600 cm^−^^1^. A weak peak, associated with the presence of hydroxyl bonds in the HA structure, can be observed at around 3400 cm^−^^1^. Peaks of both materials can be found in the composite spectra. When the HA and AG amounts were comparable (i.e., AG1HA1), peaks of both materials were present; on the other hand, when HA was larger than AG (namely, AG4HA10), agarose peaks were less relevant than HA ones.

In addition, the FT-IR spectra of the composites did not show new peaks with respect to either agarose or hydroxyapatite. Therefore, a physical mixture is obtained, and bonds are not formed between the polymer and the filler; during the preparation of the hydrogel, hydroxyapatite crystals precipitated on the polymeric backbone without any significant chemical interaction.

### 2.3. Morphology and HA Distribution

As mentioned in Section 3, the morphology of agarose aerogels and composites was investigated using FESEM analysis, whereas the composites’ HA distribution was explored by EDX.

#### 2.3.1. Agarose Aerogel Morphology

**Figure 2:**
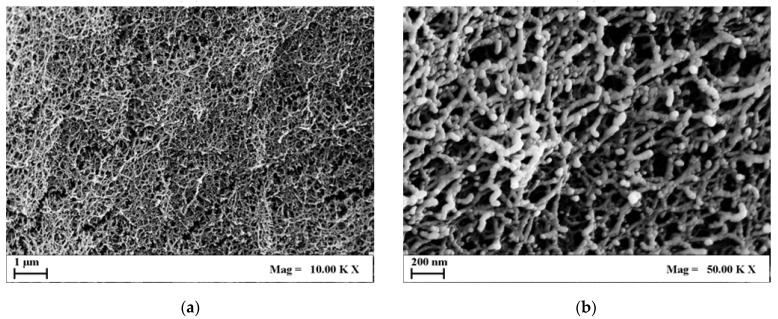
FESEM images of 1% *w*/*w* agarose aerogel cross-section at different magnifications: (**a**) 10.00 KX; (**b**) 50.00 KX.

**Figure 3:**
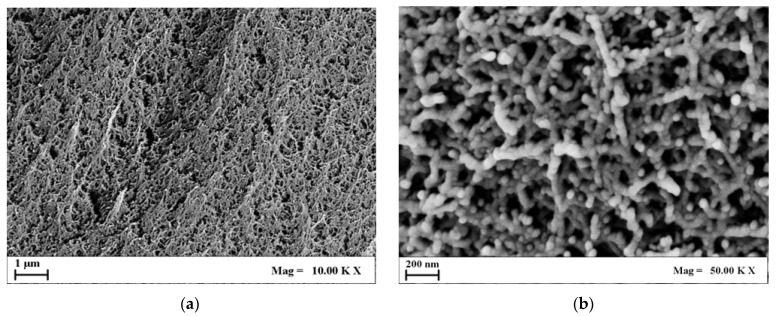
FESEM images of 4% *w*/*w* agarose aerogel cross-section at different magnifications: (**a**) 10.00 KX; (**b**) 50.00 KX.

Figure 2 and Figure 3 report a collection of FESEM images of a 1% *w*/*w* and 4% *w*/*w* agarose aerogels cross-section at different magnifications.

Comparing Figure 2 and Figure 3, it can be pointed out that 4% *w*/*w* agarose aerogels showed a closed and compact morphology. On the micrometric scale, 4% *w*/*w* aerogels lost their openness and their fiber-like structure, typical of 1% *w*/*w* agarose aerogels, which showed wider and rounder pores, and thinner fibrils. This trend could be applied to the nanoscale as well: nanopores, although more closed, were still intact even at higher polymer concentrations. In view of BTE applications, nanostructures are essential to host hydroxyapatite crystals and to favor cell adhesion and proliferation. Therefore, both structures could be eligible for bone tissue regeneration purposes.

#### 2.3.2. AG–HA Composites Morphology

**Figure 4:**
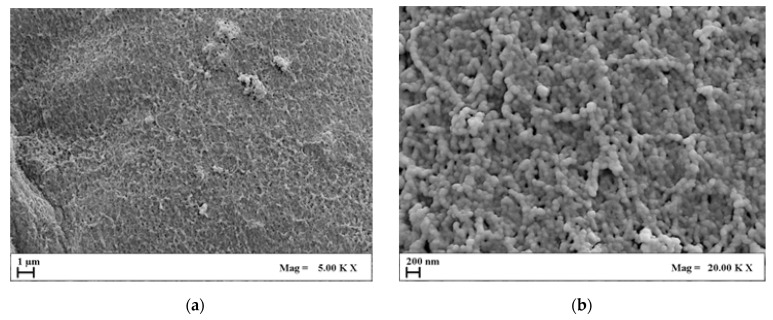
FESEM images of AG1HA1 at different magnifications: (**a**) 5.00 KX; (**b**) 20.00 KX.

Once AG aerogel morphology was assessed, HA was loaded in the aerogels. Figure 4 collects some representative FESEM images (on micro- and nanoscale) of the AG1HA1 set of composites after SC-CO_2_ drying.

Analyzing the FESEM images collected in Figure 4, it can be highlighted that aerogel morphology was partially compromised after the addition of HA. This effect became much more significant when HA content outweighed AG concentration by an order of magnitude. In these conditions, two aspects emerge: firstly, when HA concentration is greater than 1% *w*/*v*, agarose partially lost its typical morphology, especially on the microscale; secondly, nanopores, although far from the openness of pure agarose, are still partially preserved. For these reasons, the addition of large amounts of HA modified the structural integrity of the agarose, but cell attachment could still be possible. Moreover, being bones rich in hydroxyapatite, such composites could still be mimicking the original tissue on the nanoscale from a chemical and structural point of view.

**Figure 5:**
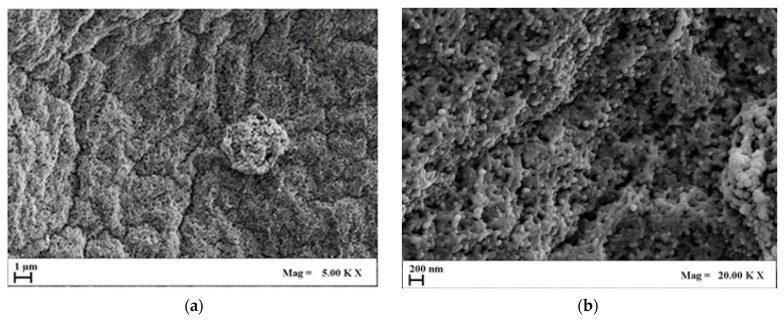
FESEM images of AG4HA1 at different magnifications: (**a**) 5.00 KX; (**b**) 20.00 KX.

The same concept could be applied to the second set of composites (i.e., those obtained using 4% *w*/*w* of AG). Figure 5 summarizes the FESEM images of AG4HA1 samples.

Figure 5 shows that the combination of AG–HA that more resembles one of pure agarose aerogel is AG4HA1, since it could still be considered as a system in which HA is dispersed onto an agarose polymeric network. For higher HA loadings (i.e., 5% and 10% *w*/*v*), the composite’s structure became progressively more closed and compact. In such cases, nanopores could still be partially observed; on the other hand, the original agarose microstructure was negatively affected. Moreover, the effect of structural closure was more evident when working at 4% *w*/*w* polymer concentration. In such cases, pure AG aerogel already showed a more compact structure with respect to 1% *w*/*w* AG, and once large amounts of HA were loaded into 4% *w*/*w* polymeric solution, the network compaction was favored.

#### 2.3.3. EDX Analysis

**Figure 6:**
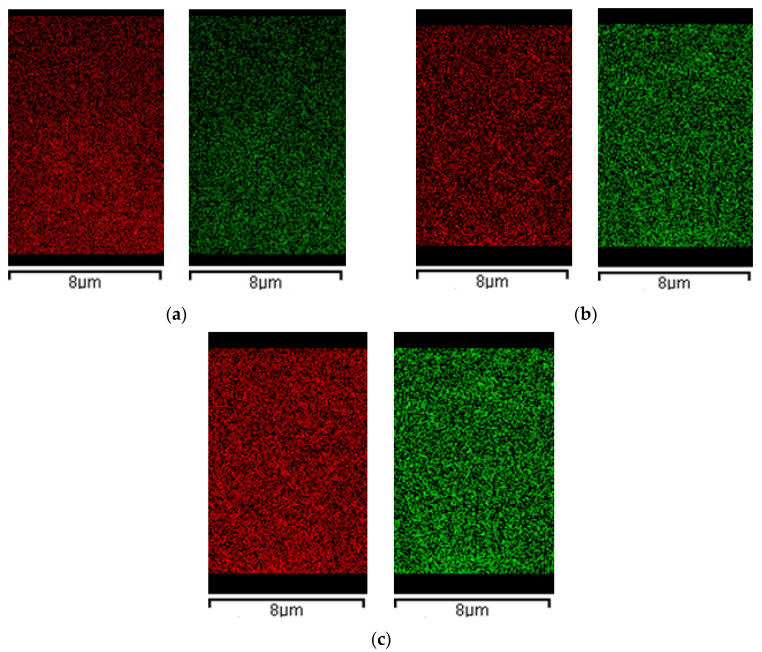
EDX spectra of calcium (red) and phosphorous (green) of: (**a**) AG1HA1; (**b**) AG1HA5; (**c**) AG1HA10.

In combination with morphological analysis, the HA distribution across the composites section is an important parameter that can prove samples’ homogeneity and regularity. Figure 6 reports EDX maps related to the calcium and phosphorous (HA main elements), of AG1HA1, AG1HA5 and AG1HA10. It proves that HA is present and equally distributed across the composites section. Moreover, the signal intensified once filler content increased. Moreover, in all three composites, HA did not form evident clusters on the microscale.

**Figure 7:**
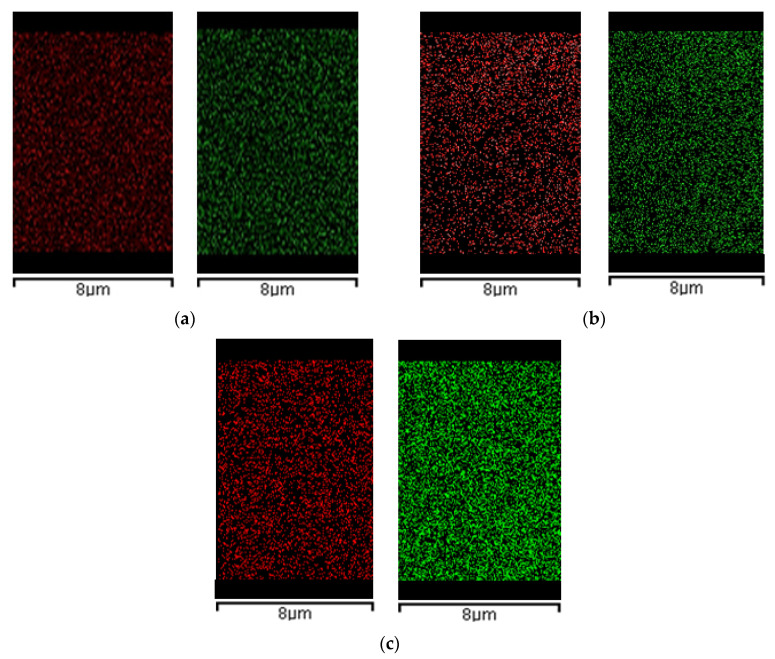
EDX spectra of calcium (red) and phosphorous (green) of: (**a**) AG4HA1; (**b**) AG4HA5; (**c**) AG4HA10.

Figure 7 is related to the collection of EDX maps of 4% *w*/*w* agarose composites. Also, in this case, HA distribution was homogeneous through the cross-sections of the composites produced.

Even though the FESEM analysis showed that HA addition can partially close the microstructure of the aerogels, as far as larger loadings were concerned, EDX outlined that cross-sections were uniform in the composition of AG–HA tested. This result could be useful for BTE applications, as homogenous structures define cell adhesion and proliferation, as well as nutrient exchange. Where the samples were morphologically and chemically heterogeneous, cells and nutrients would have preferential routes to grow and move across the scaffold, resulting, therefore, in badly developed tissues. In addition, the choice of the drying technique could have affected EDX results; as mentioned before, if the scaffold did not offer a nanostructure for HA to distribute, the filler would have clustered in the network, thus creating points of discontinuity from a compositional point of view. Moreover, the fact that phosphorous and calcium are equally distributed in the cross-section reflects that, and the supercritical CO_2_ assisted process does not interfere with atomic distribution of hydroxyapatite.

### 2.4. Compression Tests

To determine the optimum sample among the prepared AG–HA composites, morphological and chemical analyses were coupled with mechanical tests. Table 3 summarizes the values of Young’s modulus (E) for each combination of AG–HA investigated in this work.

**Figure 8:**
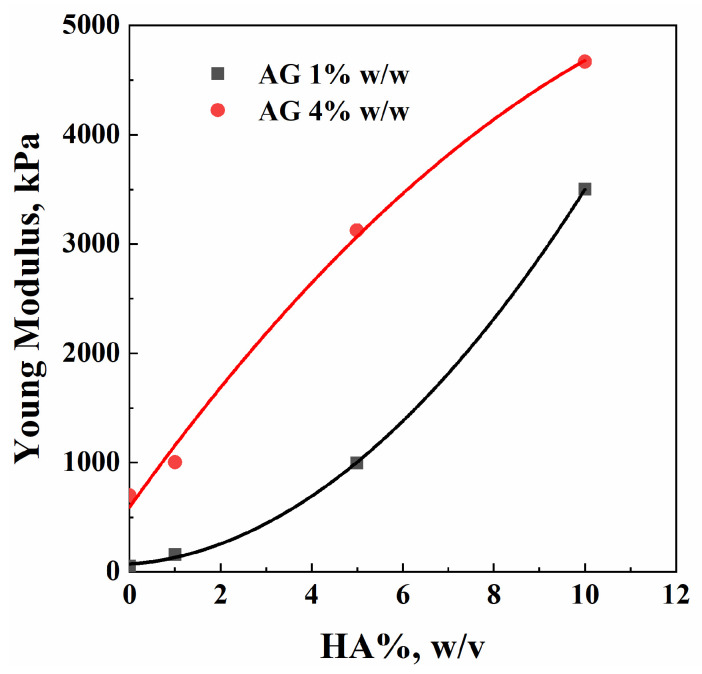
Young’s modulus vs. HA loading experimental points.

These results are represented in Figure 8 in a graphical form.

Both Table 3 and Figure 8 report changes in the composites’ mechanical behavior, which changed as a function of hydroxyapatite loading. When setting AG concentration, samples’ stiffness increased with HA concentration. Generally speaking, the same mechanical performance to compressive stress can be obtained using different combinations of AG and HA; different amounts of filler and polymer can be selected on the basis of morphology, HA distribution, and materials cost.

The values of compressive strength are not reported in Table 3 because none of the samples broke during compression tests. They showed, overall, a plastic behavior. Moreover, a bone scaffold should possess a Young’s modulus of about 100 kPa [44]; all the composites AG–HA meet this requirement, meaning that they are eligible for BTE applications. In addition, the different blends have values of E much larger than the lower boundary for BTE: not only can these composites be used as scaffolds, but they are mechanically resistant, and can withstand larger solicitations and stresses without collapsing.

## 3. Materials and Methods

### 3.1. Materials

Agarose (Type I-A, low electroendosmosis EEO) was purchased from Sigma Aldrich (St. Louis, MO, USA). Hydroxyapatite Ca_10_(PO_4_)_6_(OH)_2_ (<200 nm) was used as purchased from Sigma Aldrich (St. Louis, MO, USA). Ethanol absolute anhydrous was bought from Carlo Erba Reagenti (Chaussée du Vexin, France). CO_2_ (99.9% pure) was supplied by Morlando Group Srl. (Torre Annunziata, Italy). Distilled water was produced in a laboratory using a distillation tower.

### 3.2. Methods

#### 3.2.1. Hydrogel Preparation

Firstly, AG hydrogels were prepared by dissolving the polymeric powder in distilled water to obtain 1% *w*/*w* and 4% *w*/*w* polymer concentration. Then, once the suspension was heated up to 85 °C to guarantee agarose solubilization, it was poured into steel molds and kept at room conditions until complete gelation (hydrogel formation). AG–HA composites were prepared, adding HA (i.e., 1, 5, 10% *w*/*v*) to the heated solution, with either 1% *w*/*w* or 4% *w*/*w* of agarose. Two whole sets of composite aerogels were prepared. (AG concentration set at 1% *w*/*w*), AG1HA1 (1% *w*/*v* HA), AG1HA5 (5% *w*/*v* HA), and AG1HA10 (10% *w*/*v* HA) belong to the first set; (AG concentration set at 4% *w*/*w*), AG4HA1 (1% *w*/*v* HA), AG4HA5 (5% *w*/*v* HA), and AG4HA10 (10% *w*/*v* HA) belong to the second one.

#### 3.2.2. Supercritical Drying

Water is not miscible with SC-CO_2_ to perform SC-CO_2_ drying; water entrapped in the gel network was exchanged with an organic solvent (in this case ethanol) to guarantee liquid extraction during supercritical drying. In such a case, a solvogel is obtained. Water was gradually removed from the gel, using a 1 h-long stepwise contact with the alcoholic solution, gradually increasing ethanol concentration (10, 30, 50, 70, 90, 100% *v*/*v*). The exchange using pure ethanol lasted overnight, about 12 h. Once the complete removal of water was ensured, solvogels were put onto a sample holder and loaded into a steel high-pressure 200 mL vessel. Supercritical drying was performed following this procedure: CO_2_, stored in liquid-vapor equilibrium, was cooled down in a refrigerating bath (Julabo, mod. ED-F35, Seelbach, Germany) set at −10 °C; then, it was brought up to the desired pressure using an HPLC high-pressure pump (Gilson, mod. 146562, Lewis Center, OH, USA). The desired temperature was reached and kept constant using heating bands installed along the line and on the high-pressure vessel. Then, SC-CO_2_ reached the high-pressure vessel and extracted the ethanol in the solvogels.

The ethanol–CO_2_ mixture was separated in a vessel at atmospheric pressure; the CO_2_ flow rate was monitored using a rotameter. The temperature was controlled using PID controllers along the line (Watlow, mod. 93, St. Louis, MI, USA) and monitored thanks to type J thermocouples, while pressure was observed using pressure gauges. A synthetic scheme of the plant-scale setup is reported elsewhere [40]. The chosen pressure and temperature were 200 bar and 40 °C (ρ_CO2_ = 0.84 g/cm^3^); the CO_2_ mass flow rate was set at 0.5 kg/h. Each drying lasted about 5 h, following the indication provided in previous works [41].

The system was brought to atmospheric pressure after the experiment, using a depressurization rate of about 3 bar/min and temperature set at 40 °C. Each experiment was carried out at least twice, to ensure process repeatability.

### 3.3. Characterizations

Prior to the morphological analysis, samples were fractured using liquid nitrogen to avoid structure deformation, and the cut sample was coated using Agar Auto Sputter Coater (mod. 108 A, Stansted, UK): the coating material was gold. Aerogel morphology was investigated using field emission scanning electron microscopy (FESEM, Carl Zeiss Supra 35, Zeiss, Oberkochen, Germany). FESEM analyses were carried out on different sections of several samples. Hydroxyapatite distribution in the aerogel section was evaluated using Energy Dispersive X-ray (EDX) analysis (mod. INCA Energy 350, Oxford Instruments, Witney, UK), searching for calcium and phosphorous signals. The samples analyzed by EDX were coated with chromium, using a turbo sputter coater (mod. K575X, EmiTech Ashford, Kent, UK).

The porosity of the samples was measured using the following procedure: scaffolds were coated using a waterproof coater; then, through Archimede’s principle, the displaced volume was measured. The mass was known and, thus, the apparent density of the composite (ρ_m_), also referred to as scaffold bulk density. Then, this value was compared with the theoretical density (ρ_p_) of the composite, under the hypothesis that the scaffold was completely closed, by knowing the mass fractions of untreated agarose and hydroxyapatite in the composite. Porosity was thus evaluated as the complement to 1 of the ratio ρ_m_/ρ_p_. To calculate the approximative value of ρ_p_, the mass fractions of HA and AG in the scaffold were considered, and their densities taken from products datasheet. Such values were 3 g/cm^3^ and 1 g/cm^3^, respectively.

Fourier transform-infrared spectroscopy (FT-IR) was used to analyze chemical bonds and the presence of functional groups within the polymeric composite. The powder to be analyzed was mixed with KBr (1:100 *w*/*w*). 100 mg of this solid mixture was compacted using a hydraulic press. Then, the sample was put in an oven, and kept at 40 °C overnight to remove humidity.

Compression tests were performed at least on three samples of the same combination of AG–HA, using a universal testing machine (Zwick Roell 2014, Ulm, Germany), choosing 6 mm/min as strain rate [45] and a load of 1 kN. Young modulus (E) was calculated from the linear part of the stress–strain curve: a deformation up to 5% was considered.

## 4. Conclusions

In conclusion, agarose–hydroxyapatite composites were successfully produced by SC-CO_2_ drying technique. The morphology and the mechanical performance of the samples were strongly influenced by the addition of the ceramic filler, which can be modulated depending on the specific biomedical application. EDX and FT-IR analyses demonstrated that the agarose network effectively hosted the filler that was homogeneously dispersed in the polymeric matrix. From a mechanical perspective, Young’s modulus of AG–HA composites meets the minimum requirement for scaffolding (100 kPa). As a result of this first general analysis, the blend agarose–hydroxyapatite could be eligible for BTE applications. The most promising candidate for BTE is the composite AG1HA10, being the compromise between high porosity (86.5%) and mechanical resistance (E = 3.5 MPa).

In perspective, the morphology of the agarose-based aerogel could be improved on the microscale using a porogen, to ensure structural openings required for nutrient exchange to the cells in view of BTE applications; moreover, biological tests could be carried out on the matter to validate cellular proliferation and differentiation, and thus, the effective application of the blend AG–HA for biomedical applications.

## Figures and Tables

**Table 1:** Agarose aerogel stability over time.

Sample	Volume Post-Drying, cm^3^	Volume after 10 Days, cm^3^	Volume after 2 Months, cm^3^
AG1	0.70	0.62	0.61
AG4	0.39	0.34	0.34

**Table 2:** AG–HA composites bulk density, theoretical density (g/cm^3^), and porosity (%).

Sample	Bulk Density, g/cm^3^	Theoretical Density, g/cm^3^	Porosity, %
AG1HA1	0.069	2	96.5
AG1HA5	0.240	2.6	91.0
AG1HA10	0.381	2.8	86.5
AG4HA1	0.153	1.4	88.5
AG4HA5	0.331	2.1	84.0
AG4HA10	0.432	2.4	82.1

**Table 3:** Young’s Modulus values of the prepared composites.

AG %, *w*/*w*	HA%, *w*/*v*	E, kPa
1	0	54
	1	158
	5	995
	10	3500
4	0	700
	1	1004
	5	3122
	10	4666

## Data Availability

Data are contained within the article.
